# Women and Night Work

**Published:** 1920-06-19

**Authors:** 


### THE PUBLIC WELL-BEING.

### Women and Night Work.

A novel objection to legislative effort which aims at limiting night work for women appears in the current issue of *Time and Tide*, the new and admirable weekly review. The Government has introduced a Bill which regulates the work of women, young persons, and children, according to the principles adopted by the Washington Convention, but *Time and Tide* seems to be opposed altogether to the distinction made between men and women. It takes the subject of night work as an example, and declares there is no better case for forbidding it to women than there is for forbidding it to men. Its argument is that all night work is unpleasant, that it should be eliminated as far as possible for both sexes but that the present proposed distinction between men and women creates an economic injustice for women by forming one more artificial barrier between the work of men and women. This is a fair point for argument, but it reduces conditions of labour to the sole, rather callous basis of economics in a somewhat extravagant championship of women's rights. It is not to be presumed that the writer of the article is opposed to the provisions which restrict the employment of women before and after child-birth, but that seems to be a fundamental consideration which covers the whole question. It is no use ignoring the physiological differences of men and women. It is, for example, far more serious that a woman should be at industrial work night after night, considered from the point of view of the children she may bear, and it would be difficult to find a medical man who, if he were asked to decide between men and women, would not decide that night work had considerably fewer dangers for men, taking it from every angle. The argument that there is a good deal of humbug about the endeavour to restrict women's work at night because babies entail night work for mothers and nurses, is specious and entirely beside the point. In the first place, there is no comparison between industrial night work and nursing; in the second place because certain night work is essentially women's work, it is no argument against limiting women's night work as far as possible; and in the third place, if babies necessarily involve night work for mothers, is it not reasonable that mothers should conserve their strength beforehand by living normal lives? There are women's duties as well as women's rights. It is logical, if unwise, to hold that women ought to be free to work at night as they please, without any legal prohibition, but not many would subscribe to the dictum of *Time and Tide* that it is "no more undesirable for one sex than for the other." Enthusiasts in the cause of women's rights frequently ignore the immovable fact of sex differences, or treat it as masculine sentimentality or economic cunning.

At the recent Brussels Health Congress in a discussion on the right of married women to work in factories—a subject far wider than the one we are now considering—the British delegates as well as French doctors at the French Academy of Medicine gave their general view that there are no ill-effects to the health of either mother or infant as a result of factory work, but a resolution was passed agreeing to discourage in every possible way except by legal measures the employment of married women. It was not surprising that the British delegates were unable to go to that length, and that they refrained from voting. At the same time, a French physician, describing the views of the French Academy, said the doctors had agreed that with certain safeguards, such as forbidding dangerous or night work, factory work would not endanger the young generation. It is to be hoped these French doctors will not be considered too old-fashioned in sticking out against night work for women.

